# Beyond the battlefield: how conflict type drives acute child malnutrition in Africa

**DOI:** 10.1186/s12889-026-27644-2

**Published:** 2026-06-18

**Authors:** Thomas Jideofor Ogbu, Rebecca Louise Jones, Debarati Guha-Sapir

**Affiliations:** 1 Association for Research on Disaster Epidemiology-ASED, Brussels, Belgium; 2 Danish Centre for Health Economics (DaCHE), University of Southern Denmark, Campusvej 55, Odense M, 5230 Denmark; 3 Université catholique de Louvain, Clos Chapelle aux Champs 30, Brussels, 1200 Belgium

**Keywords:** Acute child malnutrition, Hierarchical Bayesian beta-regression, State vs non-state conflict, Humanitarian resource allocation

## Abstract

**Background:**

Continuance of global progress toward the eradication of malnutrition in all forms requires the steering of resources to priority areas. Armed conflict is a key driver of malnutrition, yet its impact differs given the conflict intensity, duration and type. In this study, we investigate the relationship between conflict type, specifically state-based and non-state conflict, and under-5 acute malnutrition in conflict-affected African populations.

**Methods:**

We link malnutrition data from small-scale surveys held in the Complex Emergency Database, conducted across 11 conflict-affected African populations between 1999 and 2015, with conflict data from UCDP/PRIO. We estimate associations between conflict type and the prevalence of global acute malnutrition (GAM) and severe acute malnutrition (SAM) whilst controlling for relevant covariates, using Bayesian beta-regression, which allows aggregation of survey data.

**Findings:**

Holding other factors constant, non-state conflict was significantly (*p* < 0.05) associated with a higher prevalence of GAM and SAM (15.27% and 4.83%) relative to state-based conflict (9.03% and 1.26%). In addition, we find heterogeneous impacts of prior military expenditure as a share of GDP, overseas development assistance (ODA) and precipitation variability on under-5 acute malnutrition by conflict type. Namely, prior military expenditure and lower-than-normal precipitation were associated with increased malnutrition prevalence under state-based conflict, yet to a lesser degree under non-state conflict. We find a significant mitigating role of ODA on GAM, irrespective of conflict type. For SAM, ODA was more negatively associated with malnutrition prevalence under non-state conflict relative to state-based conflict.

**Interpretation:**

The study extends the current literature by finding a heterogenous relationship between conflict type and under-5 acute malnutrition, namely we estimate a significantly higher malnutrition burden in African populations affected by non-state conflict relative to state-based conflict. Our findings underscore the importance of context-specific resource planning when addressing malnutrition burden in humanitarian crises.

**Supplementary Information:**

The online version contains supplementary material available at https://doi.org/10.1186/s12889-026-27644-2.

## Introduction

Global progress toward the eradication of malnutrition in all forms, a key development goal of the 2025 World Health Assembly Global Nutrition targets and the United Nations 2030 Sustainable Development Goals, is noteworthy. Over half the world’s children under 5 live in a country witnessing reductions in wasting; for stunting this figure exceeds three quarters [1]. However, of the 39.6 million children under 5 that are expected to be missed by current efforts, 80% will be in Africa [2].

Identifying drivers of malnutrition is necessary to direct scarce humanitarian resources to priority areas. Armed conflict is a major driver of malnutrition [3–5], with approximately two thirds of malnourished children living in conflict-affected areas [6]. Among other mechanisms, armed conflict can directly or indirectly affect population health through disruptions to food production, restricted access to healthcare services and sanitation, economic instability, and forced displacement of populations to areas with overburdened services and heightened insecurity [7].

Historically, armed conflict was treated as homogeneous in the empirical literature, despite its multiple facets. More recently, conflict duration and intensity, the latter generally proxied by the number of battle-related deaths or conflict events, have been positively linked to increased prevalence of malnutrition [8, 9]. Further distinguishing by conflict typology, Acharya et al. [4] found that improvised explosive device attacks and direct fire incidents were most strongly associated with child malnutrition in Iraq. In addition, a study by Goli et al. [10] found that conflict exposure, in particular to state-based conflict as opposed to one-sided violence against civilians, was negatively associated with child weight-for-age scores. However, non-state conflict was not considered due to limited data availability.

In this study, we estimate the prevalence of under-5 acute malnutrition in conflict-affected populations across Africa, where the malnutrition burden is disproportionately concentrated. We utilise small-scale survey data at the admin-1 level, i.e. administrative boundaries of the first subnational level, from the Complex Emergency Database (CE-DAT), a previously underutilised data source. Moreover, by employing Bayesian analysis to overcome limitations in data sparsity and to improve credibility intervals, we explore heterogeneity in malnutrition estimates by conflict type, namely by state-based and non-state conflict, thus building on the existing literature. Since conflict actors are typically identifiable in real-time, understanding potential heterogeneous associations between conflict type and malnutrition burden enables more pre-emptive and targeted humanitarian responses to populations at greatest nutritional risk.

## Methods

### Data

We utilise two main sources of data; nutritional data come from CE-DAT [11] and conflict data from the Uppsala Conflict Data Programme (UCDP) / Peace Research Institute Oslo (PRIO) Armed Conflict database [12]. The variables included in our final model specification, including data sources and definitions, are given in Table 1.

**Table 1:** Model variables, data sources and definitions

Variable	Data source	Definition
Global acute malnutrition (%) [outcome]	CE-DAT	Weight-for-height 2 SD below the WHO median international reference value, standardised by age and sex [11, 13].
Severe acute malnutrition (%) [outcome]	CE-DAT	Weight-for-height 3 SD below the WHO median international reference value, standardised by age and sex, and/or the presence of bilateral oedema [11, 13].
Conflict type - State-based conflict	UCDP/PRIO	The use of armed force between two parties, of which at least one party is the government of a state.
Conflict type - Non-state conflict	UCDP/PRIO	The use of armed force between two parties, neither of which is the government of a state.
Military expenditure (share of GDP)	SIPRI	All current and capital expenditure on the armed forces, defence ministries, paramilitary forces and military space activities, but excluding current expenditures on civil defence and previous military activities, measured as a share of GDP.
Overseas Development Assistance (US $)	World Bank	The disbursement of loans made on concessional terms and grants by official agencies of the members of DAC, multilateral institutions, non-DAC countries to promote economic development and welfare in countries and territories in the DAC list of aid recipients.
SPEI – dry spell	SPEIbase	Precipitation index < -1.0 in the prior 12 months.
SPEI - normal	SPEIbase	Precipitation index between − 1.0 and 1.0 in the prior 12 months.
SPEI – wet spell	SPEIbase	Precipitation index > 1.0 in the prior 12 months.

*GDP* Gross domestic product, *DAC* Development Assistance Committee, *SPEI* Standardized Precipitation-Evapotranspiration index, *CE-DAT* Complex Emergency Database, *UCDP* / *PRIO* Uppsala Conflict Data Programme / Peace Research Institute Oslo, *SIPRI* Stockholm International Peace Research Institute

#### Nutrition data

We study the association between conflict type and malnutrition burden, as measured by under-5 acute malnutrition. We focus on acute malnutrition (wasting) due to being less studied in the existing literature versus chronic malnutrition (stunting). Wasting, i.e. low weight-for-height, is the result of a sudden lack of food availability, food quality and/or illness and poses an imminent threat to life if not treated appropriately. In contrast, stunting, i.e. low height-for-age, arises due to chronic or recurrent undernutrition [14, 15].

The nutritional status of a population is typically determined through cross-sectional household surveys run at multi-year intervals, including Demographic Health Surveys, Multi-Indicator Cluster Surveys and National Nutritional Surveys, as well as small-scale surveys conducted following the Standardised Monitoring and Assessment of Relief and Transitions (SMART) framework. In this study we collate nutritional data at the admin-1 level from cross-sectional SMART surveys held in CE-DAT. SMART surveys are a common population-based approach to estimate malnutrition in areas facing humanitarian crises which are otherwise difficult to reach. CE-DAT is a repository of small-scale SMART surveys on population health and nutrition conducted in populations facing active conflict between 1999 and 2015 [11].

From CE-DAT, we acquired data on the prevalence of under-5 global acute malnutrition (GAM) and severe acute malnutrition (SAM) in conflict-affected African populations. This corresponded to 1046 survey observations across 33 admin-1 levels and 11 countries^1^ (Additional file, Table A1 and A2). CE-DAT also reports the prevalence of under-5 moderate acute malnutrition (MAM). However, due to a high proportion of missing data (> 60%) for MAM, likely due to GAM and SAM being more standard malnutrition indicators to measure and report in humanitarian crises [16], we did not consider MAM in our analysis.

#### Conflict data

**Fig. 1:**
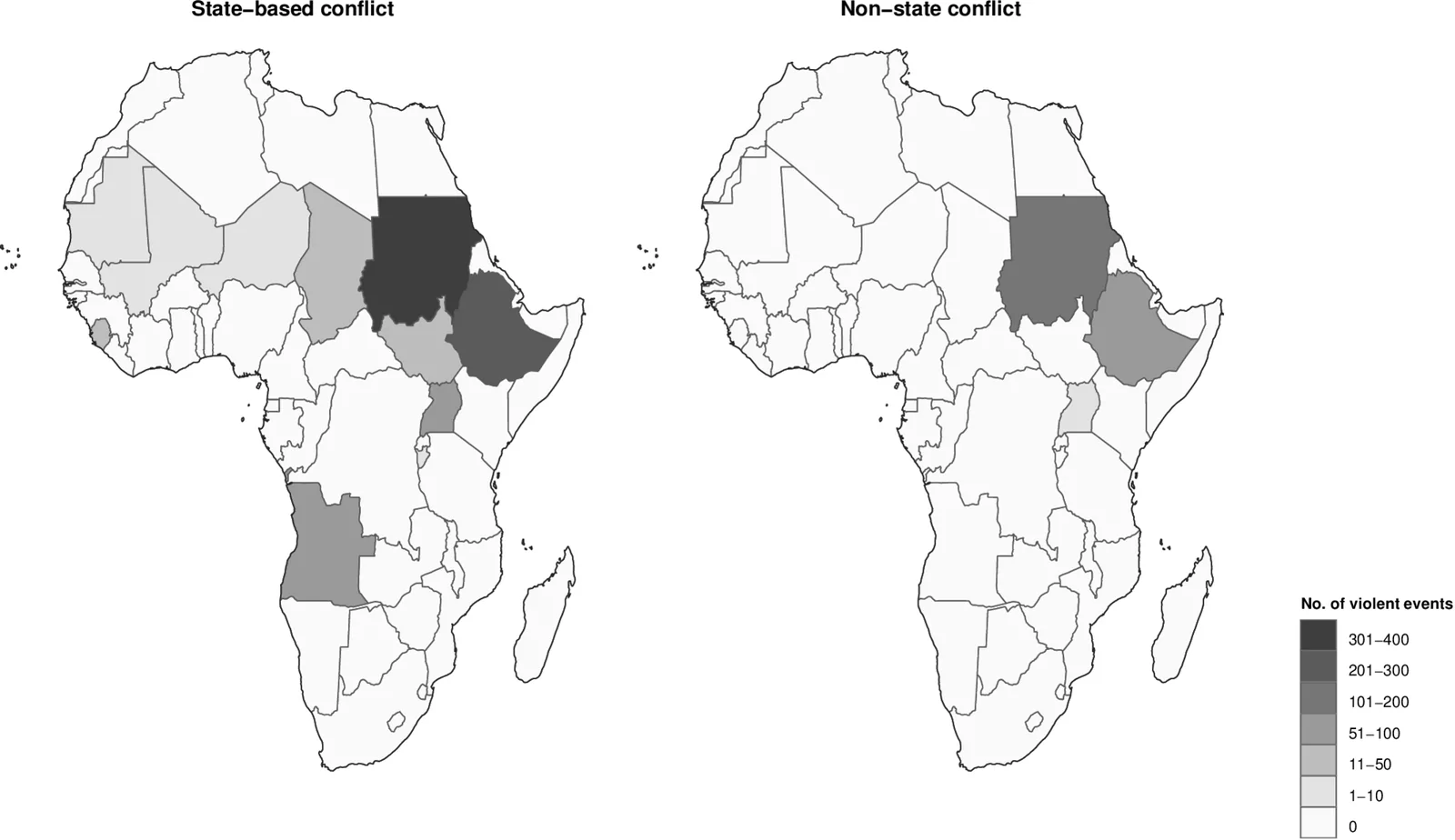
Conflict-affected African populations with available nutrition data between 1999–2015

We matched nutrition data from CE-DAT with conflict data from UCDP/ PRIO on spatial (admin-1 level) and temporal (year) dimensions. UCDP/PRIO records violent events^2^ for armed conflicts resulting in at least 25 battle-related deaths in a calendar year [17]. We acquired data on the number of violent events, conflict type (state-based conflict, non-state conflict and one-sided violence), and the date of conflict onset and conclusion. Our main explanatory variable of interest was conflict type. We observed few observations (*n* = 3) of one-sided violence and therefore, did not consider one-sided violence in our analysis. The distribution of conflict events, by conflict type, across the 11 countries in our sample are shown in Fig. 1 and detailed in Table A1 (Additional file). Sudan (*n* = 519) and Ethiopia (*n* = 313) had the highest number of violent events. We identified 18 admin-1 clusters with non-state conflict events across 3 countries: Sudan (*n* = 179), Ethiopia (*n* = 61) and Uganda (*n* = 3).

#### Covariates

We included covariates that were found to be significantly associated with under-5 acute malnutrition in a preliminary bivariate analysis (additional file, Table A3) and identified as relevant predictors in the literature. Other potential predictors: number of violent events and conflict duration, were not significant in our preliminary analysis and thus excluded from our baseline model specification.

Data on military expenditure as a share of GDP were acquired at the country level from the Stockholm International Peace Research Institute (SIPRI) database (https://www.sipri.org/databases/milex). We used this to infer the political priority given to military efforts, likely at the expense of other public services including health and education. In state-based conflicts, a higher share of GDP allocated to military expenditure is likely to reflect conflict intensity, whereas in non-state conflicts, this may loosely reflect the degree of state involvement, for instance in stabilising efforts at a local level [18].

Data on the level of overseas development assistance (ODA) received at the country-level were acquired from the World Bank (https://data.worldbank.org/) and approximated to the admin-1 level based on the number of individuals residing in each admin-1 level, or if this information was unavailable, the number of surveyed individuals. We lag military expenditure as a share of GDP and ODA by 1 year to minimise potential endogeneity due to reverse causality, i.e. where malnutrition levels influence military spending decisions or ODA allocation. This approach assumes that malnutrition in the current year does not affect military expenditure or ODA in the previous year.

The co-occurrence of climate-related hazards or disaster events during conflict exacerbates threats to malnutrition [3, 19, 20]. We control for higher- and lower-than-normal precipitation in the 12 months prior to the survey date, based on the Standardized Precipitation-Evapotranspiration index (SPEI). Data were acquired from SPEIbase (https://spei.csic.es/database.html) at a 0.5° spatial resolution and aggregated to the admin-1 level. We defined dry spells as an SPEI less than − 1.0, wet spells as an SPEI greater than 1, and normal precipitation as an SPEI between − 1.0 and 1.0, based on the classification systems adopted by Danandeh Mehr et al. [21] and Lee et al. [22]. Variability in precipitation from the norm is likely to have protracted effects on food insecurity and malnutrition given the stage of the growing season. We also considered the SPEI in the 3 and 6 months prior to the survey date, yet we did not find statistically significant correlations with malnutrition prevalence (results available on request).

### Statistical analysis

We estimated the association between conflict type and under-5 acute malnutrition using Bayesian beta-regression models, fitted separately for GAM and SAM. This approach allowed us to aggregate data from individual small-scale SMART surveys, which typically have limited sample sizes and varying sampling methods [23], to achieve greater population coverage and more representative estimates. Beta-regression is well-suited to model prevalence outcomes, since it accommodates values bounded between 0 and 1. In addition, unlike general linear models, beta-regression accommodates non-constant variance and skewed distributions, which are common in survey prevalence data from humanitarian emergencies, where prevalence is typically right-skewed, with a few extreme values.

All analyses were performed using RStudio (v4.0.2) using the packages *rjags* (v4-16) [24] HDInterval (v0.2.4) [25] and ggplot2 (v3.4.4) [26].

#### Model specification

Our outcome variables (prevalence of GAM and SAM) were transformed into a (0,1) scale required by the beta distribution, ensuring extreme values approached but did not equal 0 or 1. We modelled the relationship between conflict type and malnutrition prevalence, adjusting for pre-specified confounders using the logit link function:

$$\begin{aligned}\:logit\left({\mu\:}_{i}\right)=\:\theta\:&+{\beta\:}_{1}Conflic{t}_{i}+\:{{\beta\:}_{2}MilltaryExp}_{it-1}\\&+{\:\beta\:}_{3}{ODA}_{it-1}+{\beta\:}_{4}{Dry}_{i}+{\beta\:}_{5}Wet_{i}\\&+{\:\beta\:}_{6}\left(Conflic{t}_{i}{*MilltaryExp}_{it-1}\right)\\&+{\beta\:}_{7}\left(Conflic{t}_{i}{*ODA}_{it-1}\right)+\:{\:\beta\:}_{8}\left(Conflic{t}_{i}{*Dry}_{i}\right)\\&+\:{\:\beta\:}_{9}\left(Conflic{t}_{i}{*Wet}_{i}\right)+\:{\gamma\:1}_{i}\end{aligned}$$

where, $\:{\mu\:}_{i}$ is the prevalence of GAM or SAM for observation * i*;$\:\:\theta\:$ is the baseline malnutrition prevalence under state-based conflict (reference category), all else equal; $\:{\beta\:}_{1}$ captures the effect of non-state conflict relative to state-based conflict; $\:{\beta\:}_{2}$ - $\:{\beta\:}_{5}$ reflect the effects of military expenditure as a share of GDP (lagged by one year), ODA (lagged by one year), dry and wet spells (versus a normal SPEI) in the preceding 12 months of the survey date, respectively under state-based conflict. State-based conflict served as the reference category due to having more observations.

To test heterogeneity in the association between our model covariates and under-5 acute malnutrition by conflict type, we included interaction terms with conflict type. Hence $\:{\beta\:}_{6}$ - $\:{\beta\:}_{9}$ can be interpreted as the effect of each covariate under non-state conflict, relative to state-based conflict. We included a random effect term to account for clustering within admin-1 areas and unobserved time-invariant differences between admin-1 areas, for instance in baseline vulnerability and service access. As a robustness check, we fit a simplified model including only conflict type. We then systematically omitted one admin-1 cluster at a time to verify that no single cluster disproportionately influenced our estimates (Additional file, Figure A1). In addition, we estimated our model using only observations from clusters having both state-based and non-state conflict events (Additional file, Table A4). The direction of the results remained consistent, indicating that the results were robust to the exclusion of any single cluster or subsets of clusters. In addition, we tested the sensitivity of our results to an alternative specification of military expenditure and ODA using a two-stage approach in line with Dominici et al. [27] and Bellemare et al. [28] in which observed, lagged values were used to predict contemporaneous values that were then entered into the beta-regression.

We assigned weakly informative priors to the model parameters, with normal distributions centred at zero. For the conflict type parameter, we used an informative prior (normal distribution with mean − 0.1) to improve computational efficiency and convergence, given the complexity of our hierarchical model structure. We estimated the model using observations with data available for all variables (i.e. complete case analysis). A missing data diagnosis confirmed data were missing completely at random, indicating minimal risk of bias with this approach [29].

#### Model estimation and diagnostics

Posterior distributions were estimated using Markov Chain Monte Carlo (MCMC) sampling with three chains and 500,000 iterations. To ensure a stable sample with minimal autocorrelation, we discarded the first 250,000 samples as burn-in and retained every 5th draw thereafter (i.e. thinning rate of 5). We assessed model convergence by the Geweke and Brooks-Gelman-Rubin diagnostics, achieving a Gelman-Rubin R-hat value of 1.0, which indicates successful convergence. Visual inspection of trace plots confirmed a good mix of MCMC chains (Additional file, Figure A2 and A3). Model fit, i.e. how well the model represents patterns in the observed data, was assessed using the posterior predictive check, where values close to 0.50 indicate excellent model fit. We derived Bayesian *p*-values of 0.50 for GAM and 0.48 for SAM (Additional file, Figure A4).

We conducted several robustness checks. To ensure posterior estimates were stable, and to test for collinearity, we considered a range of prior distributions [30] (Additional file, Figure A5). We did not find a pronounced difference in the posterior estimates under different priors and therefore did not suspect collinearity. We also examined potential outliers using the inverse conditional posterior ordinate approach, which indicated no influential outliers (values ranged between 0 and 2 for both GAM and SAM).

## Results

### Descriptive statistics

Our analysis included a total of 1,046 observations for GAM and 951 for SAM. In general, we see a balance of covariates across the groups (Table 2). State-based conflict accounted for over 70.0% of observations. Military expenditure as a share of GDP in the prior year was on average 3.6% and ODA received in the prior year ranged from 10.7 million (SAM) to 12.0 million (GAM). Most observations (~ 83.0%) were in areas that experienced dry spells (SPEI < − 1.0) in the 12 months preceding survey initiation, whereas 2.3% − 3.5% were in areas that experienced wet spells (SPEI > 1.0).

**Table 2:** Descriptive statistics

Variable	GAM			SAM		
	Obs.	Mean	Std. dev	Obs.	Mean	Std. dev
Conflict type - State-based conflict	803	0.768	-	728	0.766	-
Conflict type - Non-state conflict	243	0.232	-	223	0.234	-
Military expenditure [share of GDP]	1046	0.036	0.019	951	0.036	0.019
Overseas Development Assistance ($)	1046	11,983,134	32,014,542	951	10,659,249	27,342,716
SPEI – dry spell	868	0.830	-	792	0.833	-
SPEI - normal	141	0.135	-	37	0.144	-
SPEI – wet spell	37	0.035	-	22	0.023	-

*GDP* Gross domestic product, *SPEI* Standardized Precipitation-Evapotranspiration index, *GAM* Global acute malnutrition, *SAM* Severe acute malnutrition

### Baseline specification

Table 3 presents the beta-regression output for our baseline model specification. The constant can be interpreted as the baseline prevalence of GAM or SAM under state-based conflict with normal precipitation in the preceding 12 months, holding all other covariates at their mean values.

#### State-based conflict

Under state-based conflict, the baseline prevalence of GAM was 9.03% and the baseline prevalence of SAM was 1.26%. A one standard deviation (SD) increase in military expenditure as a share of GDP in the prior year was associated with significantly higher under-5 acute malnutrition, with log-odds of 0.118 for GAM and 0.187 for SAM (*p* < 0.05), equivalent to a 1.13 and 0.25% points increase in baseline prevalence.

Dry spells in the 12 months preceding survey initiation were significantly associated with a higher prevalence of GAM (log-odds: 0.218, *p* < 0.05), equivalent to a 2.20% points increase. In contrast, we found no significant association between wet spells and under-5 acute malnutrition. Higher ODA received in the prior year was associated with a lower prevalence of GAM (log odds: -0.081, *p* < 0.05), yet only equivalent to a 0.71% points decrease.

**Table 3:** Beta-regression outputs and calculated average prevalences

	GAM			SAM		
	Coef.	HDI (lower, upper)	Prev. (%)	Coef.	HDI (lower, upper)	Prev. (%)
Constant	-2.405*	-2.634, -2.162	9.03	-4.377*	-4.632, -4.101	1.26
Military expenditure of GDP	0.118*	0.069, 0.168	10.16	0.187*	0.106, 0.268	1.51
ODA	-0.081*	-0.130, -0.033	8.32	-0.035	-0.115, 0.046	1.21
SPEI - normal [reference]	-	-	-	-	-	-
SPEI - dry	0.218*	0.070, 0.364	11.23	0.118	-0.122, 0.357	1.41
SPEI - wet	0.043	-0.193, 0.284	9.64	-0.116	-0.608, 0.374	1.12
Conflict type (1 = non-state conflict)	0.526*	0.191, 0.846	15.27	1.347*	0.736, 1.984	4.83
*Interaction with conflict type*						
x Military expenditure of GDP	-0.176*	-0.274, -0.080	14.42	-0.280*	-0.447, − 0.111	4.40
x ODA	-0.045	-0.115, 0.023	13.47	-0.161*	-0.272, -0.051	3.97
x SPEI - dry	-0.101	-0.395, 0.191	17.17	-0.317	-0.875, 0.230	3.96
x SPEI - wet	0.169	-0.319, 0.655	19.32	1.139*	0.177, 2.116	13.44

Coefficients are reported as the posterior mean with the lower and upper Highest Density Interval (HDI) reported. Prev, denotes the average prevalence of global acute malnutrition (GAM) or severe acute malnutrition (SAM). The constant denotes the reference level of under-5 acute malnutrition. Military expenditure as a share of GDP and official development assistance (ODA) are lagged by one year in the model. The standardized precipitation evapotranspiration index (SPEI) is in the 12 months prior to the survey date

*p < 0.05

#### Non-state conflict

Populations affected by non-state conflict had significantly higher under-5 acute malnutrition relative to those affected by state-based conflict, with log-odds of 0.526 for GAM and 1.347 for SAM (*p* < 0.05), equivalent to a 6.24 and 3.57% points increase in baseline prevalence respectively.

We included conflict type interactions to explore heterogeneity in the relationships between under-5 acute malnutrition and our selected covariates by state-based and non-state conflict. Military expenditure as a share of GDP in the prior year showed a significantly weaker association with GAM and SAM under non-state conflict relative to state-based conflict. We find that ODA received in the prior year more strongly reduced SAM under non-state conflict relative to state-based conflict. Lastly, wet spells in the preceding 12 months had a significantly stronger impact on SAM in populations affected by non-state conflict relative to state-based conflict, equating to a notable 8.44% points increase in prevalence relative to normal precipitation levels of under non-state conflict.

### Sensitivity analysis

We tested the sensitivity of our results to predicted, contemporaneous values of military expenditure and ODA (Additional file, Table A5). Our results remained largely consistent with our baseline model specification, with the exception that the coefficient on conflict type for GAM became insignificant, and heterogeneity in the association between military expenditure and under-5 acute malnutrition by conflict type increased.

In addition, we specified a model including only conflict type, without controls (Additional file, Table A5). We find a positive association between conflict type and prevalence of GAM (*p* < 0.05), consistent with our baseline model specification, albeit with reduced magnitude. However, the coefficient on SAM became insignificant.

## Discussion

Despite global progress toward the eradication of malnutrition in all forms, pockets of high disease prevalence persist in a few low- and middle-income countries. Often, these countries are affected by armed conflict [31]. We find that non-state conflict was associated with a markedly higher prevalence and severity of under-5 acute malnutrition than state-based conflict across 11 conflict-affected populations in Africa. This builds on existing empirical evidence that associates high intensity and protracted conflict [8, 9] with greater malnutrition risk, and extends research by Goli et al. [10] exemplifying heterogeneity in acute child malnutrition burden by conflict type.

The prevalence of GAM in children under-5 was estimated to be 9.03% in populations affected by state-based conflict and 15.27% in populations affected by non-state conflict. Heterogeneity in the prevalence of SAM by conflict type was even more notable; the estimated prevalence of SAM in children under-5 was 2.17% under state-based conflict and 5.31% under non-state conflict. A higher malnutrition burden under non-state conflict may reflect the typical nature of non-state conflict: often involving the destruction of and control over agriculture as military tactics; and characterised by localised, fragmented and protracted fighting [32] which is likely to obstruct civilian mobility and access to food and other essential services [33]. Although, we cannot draw causal inference from this study, this finding has implications for resource planning in conflict settings, namely further resources may be directed to areas affected by non-state conflicts where a higher malnutrition burden is possible.

We also found significant variation by conflict type in the slope of the relationships between under-5 acute malnutrition and model covariates: military expenditure as a share of GDP, ODA and higher-than-normal precipitation. In this study we infer the political priority given to ongoing conflict by the share of GDP directed to military efforts, likely at the expense of other public sectors. Greater military expenditure as a share of GDP was associated with increased prevalence of under-5 acute malnutrition in populations affected by state-based conflict, which is in line with a study by Scanlan and Jenkins [34] finding a negative relationship between food security and the level of military expenditure and military arms imports. However, the association was significantly weaker under non-state conflict, consistent with state military expenditure having less direct relevance to conflicts in which the state is not a primary actor.

Higher ODA received by a country in the prior year was associated with a reduced prevalence of GAM in populations affected by state-based conflict. This is consistent with existing literature suggesting that aid interventions, e.g. nutritional interventions [35], peace operations [36] and unconditional cash transfers [37] can reduce malnutrition in conflict settings. For SAM, we do not find a statistically significant association with ODA under state-based conflict, yet under non-state conflict, we find a significantly stronger association. One possible explanation is that aid access, targeting, or service disruption may vary by conflict type, impacting the effectiveness of treatment of SAM [38]. These findings therefore underscore the value of more context-specific humanitarian approaches. In addition, a further exploration of the influence of ODA by type and distribution would help to better inform such approaches.

Lastly, our findings contribute to a body of literature linking climate variability with food insecurity in conflict settings [3, 19, 39, 40] by assessing the joint association of armed conflict and precipitation variability with under-5 acute malnutrition. We find different impacts of dry and wet spells across conflict types: dry spells were associated with increased GAM prevalence under state-based conflict, and to a lesser degree under non-state conflict, whereas wet spells had a significantly stronger association with SAM under non-state conflict relative to state-based conflict. These findings should, however, be interpreted in light of the possibility that climate conditions themselves affect conflict dynamics, making it difficult to isolate distinct mechanisms linking climate variability, conflict, and malnutrition. In addition, our results are likely context-specific, partly reflecting the vulnerability of specific populations to climate extremes [41–43]. In our sample, populations affected by non-state conflict were predominantly in Sudan, a country less prone to wet spells. Wet spells in the 12 months preceding survey initiation may therefore represent a more extreme departure from the climatic norm, contributing to the observed association between wet spells and SAM under non-state conflict.

### Limitations

Utilising data at the admin-1 level we achieve greater precision in our model estimates versus country-level estimates. However, our study remains prone to ecological fallacy, i.e. inferring individual effects from group effects. Limitations inherent to small-scale SMART surveys, including data availability and selection bias arising from non-random sampling frames, could not be easily circumvented. This meant that some admin-1 level clusters were over-represented in our sample, for instance Darfur and Oromia (Additional file, Figure A1) which may have skewed our model estimates. Despite this, no single subset of clusters drove the results. In addition, although our random-effects specification is a pragmatic way to account for spatial heterogeneity across admin-1 areas given data constraints, estimates may still be biased if unobserved area characteristics are correlated with both conflict type and malnutrition outcomes.

In this study, we do not assess temporal heterogeneity over the study period. Because SMART surveys are typically cross-sectional, they capture only a snapshot of the malnutrition burden at a given point in time. We were therefore unable to assess how associations vary over time by conflict type, including whether coordination of recovery efforts post-conflict differed systematically, or to examine changes in under-5 acute malnutrition relative to pre-conflict levels, where prevalence may already have been high. Our findings should therefore be interpreted as cross-sectional associations.

We attempt to address potential endogeneity due to reverse causality in our model by utilising lagged values for military expenditure as a share of GDP and ODA. However, it is likely that some residual bias remains. Namely, despite our model exhibiting a high goodness of fit, we may still suffer omitted variable bias. Hence, future work should look to control for additional covariates for instance access to clean water, forced displacement, mother’s education and domestic violence. In addition, although under-5 malnutrition can serve as a proxy for broader population health, the results of the study cannot be easily generalised to other age groups and should be interpreted in light of the limited geographic coverage, and as associative, rather than causal evidence.

## Concluding remarks

The eradication of malnutrition in all its forms demands context-specific planning and delivery of scarce resources. This study extends our understanding of the heterogeneous impacts of armed conflict on under-5 acute malnutrition by conflict type. We find that non-state conflict was associated with a disproportionately higher burden of under-5 acute malnutrition across 11 African populations than state-based conflict, potentially reflecting the localised, protracted nature of non-state conflict. Moreover, we find heterogeneity in the impact of other predictors of malnutrition by conflict type. Dry spells were associated with higher GAM under state-based conflict, whereas wet spells were more strongly associated with SAM under non-state conflict. Military expenditure as a share of GDP was associated with a higher prevalence of GAM and SAM under state-based conflict, yet the association was significantly weaker under non-state conflict, consistent with a lesser role of the state. Conversely, ODA was associated with a lower prevalence of GAM, irrespective of conflict type, yet the association was insignificant for SAM and requires further exploration.

Collectively, these findings highlight the limitations of a one-size-fits-all approach to humanitarian response during conflict and emphasise the importance of considering conflict type when planning context-specific resource allocation. Without more targeted approaches, large populations of children may remain beyond the reach of global commitments to eradicate malnutrition.

## Supplementary Information

Supplementary Material 1.

## Data Availability

The data analysed in this study is available on request. However, researcher interested in the raw dataset can contact the following: for the full survey results obtained from CE-DAT, should contact the Center for Research on the Epidemiology of Disasters for nutrition data. The conflict type data are publicly available online (https://ucdp.uu.se/downloads) . Data on military expenditure as share of GDP is available online (https:/data.worldbank.org/indicator/MS.MIL.XPND.GD.ZS) on World Bank Group Database. The standardized precipitation evaporation index data is publicly available https://spei.csic.es/database.html.

## Abbreviations

CE-DAT: Complex Emergency Database
GAM: Global acute malnutrition
GDP: Gross domestic product
MAM: Moderate acute malnutrition
MCMC: Markov chain Monte Carlo
ODA: Overseas development assistance
PRIO: Peace Research Institute Oslo
SAM: Severe acute malnutrition
SIPRI: Stockholm International Peace Research Institute
SMART: Standardised Monitoring and Assessment of Relief and Transitions
SPEI: Standardized Precipitation-Evapotranspiration Index
UCDP: Uppsala Conflict Data Programme (UCDP) /
WHZ: Weight-for-height z-score

## Footnotes

1 The 11 countries with available nutrition data were: Angola, Burundi, Chad, Ethiopia, Mali, Mauritania, Niger, Sierra Leone, South Sudan, Sudan, Uganda.

2 UCDP categorise a violent event as: “an individual incident of lethal violence occurring at a given time and place”.
