# Supplementary material for: Beyond the battlefield: how conflict type drives acute child malnutrition in Africa
Source: BMC Public Health. 2026 Jun 18;26:2783. doi: 10.1186/s12889-026-27644-2 (PMC13637298; doi:10.1186/s12889-026-27644-2)
Supplement: Supplementary file 1 [file 12889_2026_27644_MOESM1_ESM.docx]

# **Additional File**

Table A 1:Number of observations by country and region

| Country | Region | Admin1 | State-based conflict | Non-state conflict | Total no. of observations (surveys) |
| --- | --- | --- | --- | --- | --- |
| Angola | Middle Africa | 6 | 62 | 0 | 62 |
| Burundi | Eastern Africa | 1 | 2 | 0 | 2 |
| Chad | Middle Africa | 2 | 31 | 0 | 31 |
| Ethiopia | Eastern Africa | 5 | 252 | 61 | 313 |
| Mali | Eastern Africa | 1 | 5 | 0 | 5 |
| Mauritania | Western Africa | 1 | 2 | 0 | 2 |
| Niger | Western Africa | 2 | 7 | 0 | 7 |
| Sierra Leone | Western Africa | 2 | 12 | 0 | 12 |
| South Sudan | Northern Africa | 2 | 30 | 0 | 30 |
| Sudan | Northern Africa | 5 | 340 | 179 | 519 |
| Uganda | Eastern Africa | 8 | 60 | 3 | 63 |

The number of observations per year ranges from 1 in 1999 and 2015, to 162 (GAM) and 147 (SAM) in 2005. While the average prevalence ranges from 6.88 (GAM) and 0.48 (SAM) in 2010 to 24.2 (GAM) and 5.4 (SAM) in 2000.

Table A 2:Distribution of observations, admin1, and average prevalence of GAM and SAM by year

|  | Global Acute Malnutrition | | | Severe Acute Malnutrition | | |
| --- | --- | --- | --- | --- | --- | --- |
| Year | No. admin1 | No.obs | Av.Prev | No. admin1 | No.obs | Av.Prev |
| 1999 | 1 | 1 | 10 | 1 | 1 | 2.4 |
| 2000 | 11 | 73 | 24.2 | 11 | 71 | 5.4 |
| 2001 | 8 | 69 | 16.63 | 8 | 67 | 2.71 |
| 2002 | 9 | 71 | 17.28 | 9 | 67 | 2.61 |
| 2003 | 8 | 31 | 14.33 | 8 | 30 | 2.57 |
| 2004 | 9 | 100 | 15.06 | 9 | 91 | 2.86 |
| 2005 | 9 | 162 | 13.06 | 9 | 147 | 1.56 |
| 2006 | 8 | 81 | 15.13 | 8 | 71 | 1.75 |
| 2007 | 8 | 107 | 14.4 | 8 | 88 | 1.72 |
| 2008 | 10 | 144 | 11.97 | 10 | 125 | 1.55 |
| 2009 | 3 | 58 | 12.78 | 3 | 53 | 1.67 |
| 2010 | 2 | 32 | 6.88 | 2 | 29 | 0.48 |
| 2011 | 4 | 32 | 10.34 | 4 | 32 | 2 |
| 2012 | 4 | 23 | 10.43 | 4 | 22 | 1.33 |
| 2013 | 4 | 37 | 9.87 | 4 | 35 | 1.07 |
| 2014 | 4 | 24 | 12.85 | 4 | 21 | 2.13 |
| 2015 | 1 | 1 | 16.1 | 1 | 1 | 2.4 |

## **Sensitivity Analysis and Robustness check**

Table A3 shows that the variables of interest; military expenditure as a share of GDP, ODA, and SPEI are significantly associated with both Global Acute Malnutrition and Severe Acute Malnutrition. Although the association between conflict type alone and SAM is not significant, the direction of the association remains consistent in the full model.

Table A 3: Posterior estimates and density intervals for univariate Bayesian Beta mixed effect models

|  | Global Acute Malnutrition | | | Severe Acute Malnutrition | | |
| --- | --- | --- | --- | --- | --- | --- |
|  | Mean | Lower  HDI | Upper HDI | Mean | Lower  HDI | Upper HDI |
| Conflict Type | 2.723 | 2.466 | 3.016 | 0.103 | -0.074 | 0.28 |
| Military exp. of GDP | 0.094 | 0.046 | 0.142 | 0.22 | 0.144 | 0.297 |
| ODA | -0.101 | -0.14 | -0.061 | -0.096 | -0.162 | -0.029 |
| SPEI dry | 0.253 | 0.132 | 0.376 | 0.346 | 0.147 | 0.545 |
| SPEI wet | -0.14 | -0.349 | 0.069 | -0.116 | -0.554 | 0.322 |

We conducted an additional sensitivity analysis, using only data from the overlapping countries. The results (Table A4) show that the direction of the association for main effects remained unchanged, although, the magnitudes were higher in this subset. This pattern reflects the fact that all variation in conflict type is contained within the subset of the overlapping countries, whereas the remaining observations in the full dataset consist only of state-based conflict, which impact the overall effect size. Most importantly, the consistency in direction across both models indicates that the overlapping countries are not driving the association; rather it provides the useful variation, and the inclusion of the observations with only state-based conflict reduces the magnitude without altering the underlying relationship.

Table A 4:Posterior estimates and high-density interval for overlapping admin1 (3 countries)

|  |  | Global Acute Malnutrition | | | Severe Acute Malnutrition | | |
| --- | --- | --- | --- | --- | --- | --- | --- |
|  |  | Mean | Lower HDI | Upper HDI | Mean | Lower HDI | Upper HDI |
| State-based conflict | Constant | -2.221 | -2.509 | -1.952 | -4.119 | -4.432 | -3.772 |
|  | Military exp. of GDP | 0.144* | 0.095 | 0.192 | 0.133* | 0.05 | 0.216 |
|  | ODA | -0.073* | -0.123 | -0.023 | -0.029 | -0.115 | 0.057 |
|  | SPEI normal [ref] | - | - | - | - | - | - |
|  | SPEI dry | 0.216* | 0.07 | 0.361 | 0.126 | -0.121 | 0.371 |
|  | SPEI wet | 0.013 | -0.229 | 0.256 | -0.153 | -0.648 | 0.338 |
|  | Conflict type[=1, non-state conflict] | 0.482* | 0.174 | 0.797 | 0.693* | 0.103 | 1.303 |
| Non-state conflict | Conflict type*military | -0.166* | -0.274 | -0.059 | -0.031* | -0.215 | 0.151 |
|  | Conflict type*ODA | -0.081* | -0.143 | -0.018 | -0.177* | -0.282 | -0.072 |
|  | Conflict type*dry | -0.209 | -0.478 | 0.061 | -0.177 | -0.707 | 0.355 |
|  | Conflict type*wet | 0.322 | -0.105 | 0.749 | 0.919 | 0.032 | 1.826 |

** significant at 0.05. ODA and Military Expenditure per GDP are lagged

Table A5 presents the posterior estimates and density intervals for Model A (conflict‑only model) and Model B (two‑stage lagged model). In the conflict‑only model (Model A), conflict type is significantly associated with Global Acute Malnutrition, but not with Severe Acute Malnutrition.

Table A 5: Posterior estimates and density intervals

|  |  |  | Global Acute Malnutrition | | | Severe Acute Malnutrition | | |
| --- | --- | --- | --- | --- | --- | --- | --- | --- |
|  |  |  | Coef. | HDI  (lower, upper) | Prev.  (%) | Coef. | HDI  (lower, upper) | Prev.  (%) |
| Model A | Conflict Only Model | Constant | -2.289 | -2.506, -2.08 | 10.14 | -4.234 | -4.474, -3.981 | 1.45 |
|  |  | Non-state | 0.137* | 0.03,0.243 | 11.65 | 0.101 | -0.076,0.280 | 1.6 |
|  | Two stages: |  |  |  |  |  |  |  |
|  |  | Constant | -2.315* | -2.55, -2.080 | 9.88 | -4.209* | -4.457, -3.938 | 1.49 |
| Model B | State based | Military Expenditure of GDP | 0.183* | 0.105, 0.263 | 11.86 | 0.295* | 0.164, 0.427 | 1.99 |
|  |  | ODA | -0.084* | -0.133, -0.034 | 9.08 | -0.036 | -0.12, 0.046 | 1.43 |
|  |  | SPEI – dry | 0.216* | 0.07, 0.36 | 12.26 | 0.119 | -0.12, 0.357 | 1.67 |
|  |  | SPEI - wet | 0.064 | -0.181, 0.309 | 10.53 | -0.12 | -0.617, 0.373 | 1.32 |
|  | Non-state based | Non-state | 0.159 | -0.091, 0.422 | 11.58 | 0.711* | 0.196, 1.232 | 3.03 |
|  | Interaction | Military Expenditure of GDP | -0.359* | -0.563, -0.16 | 9.71 | -0.596* | -0.953, -0.244 | 2.24 |
|  |  | ODA | -0.08 | -0.201, 0.039 | 9.83 | -0.273* | -0.46, -0.086 | 2.22 |
|  |  | SPEI – dry | -0.106 | -0.413, 0.197 | 12.93 | -0.331 | -0.915, 0.256 | 2.45 |
|  |  | SPEI - wet | 0.183 | -0.309, 0.702 | 14.82 | 1.165* | 0.137, 2.196 | 8.60 |

** significant at 0.05

Impact of admin level

We assess the influence of administration levels on the relationship between conflict and malnutrition. To achieve this, we systematically refitted the model by omitting one administrative level at a time – model with only conflict type and but excludes other moderating variables. The omission of Oromo or West Darfur shows a considerable shift in the posterior estimates for both the GAM and SAM model. Specifically, the omission of West Darfur from GAM and SAM models indicates significant posterior estimates – which is similar to the result obtained with all the administration levels - the effects are not observed with the omission of any other clusters.

**Figure A 1: Posterior estimates and highest density interval for omission of clusters.**

## Stationarity

The trace plot indicates the presence of a good mix between the 3 chains, hence, stationarity.

Figure A 2: Trace plots of Global Acute Malnutrition Model.

** Where the covariates betacov[1] – betacov[6] are ODA, Military expenditure per GDP, Military expenditure per GDP*Conflict type, ODA*conflict type, Drought*conflict type, Wet*conflict type, respectively. Spei_a[2] and Spei_a[3] are drought and wet respectively.

The trace plots indicate a good mix between the 3 chains and with Rhat value close to 1, hence, stationarity.

Figure A 3:Trace plots of Severe Acute Malnutrition Model

** Where the covariates betacov[1] – betacov[6] are ODA, Military expenditure per GDP, Military expenditure per GDP*Conflict type, ODA*conflict type, Drought*conflict type, Wet*conflict type, respectively. Spei_a[2] and Spei_a[3] are drought and wet respectively.

## Goodness of fit test

We used posterior predictive checks plot to test for the goodness of fit of the model. The observed values are plotted against the values generated by the model. The posterior p-values were also calculated and a posterior p-value close to 0.5 is an indication that the model fits the data well.

The posterior predictive value close to 0.5 indicates that the model is a good fit for the data

Figure A 4 Plot of posterior predictive checks for the Global Acute Malnutrition and Severe Acute Malnutrition Models

| Global Acute Malnutrition | Severe Acute Malnutrition |
| --- | --- |
|  |  |

## Test for outliers

We plotted the inverse conditional posterior ordinate for the observations and the plots indicate that the inverse-cpo values are less than 40, hence, an indication of no extreme value.

Figure A 5:Plot of inverse conditional posterior ordinate for the Global Acute Malnutrition and Severe Acute Malnutrition Models.

| Global Acute Malnutrition | Severe Acute Malnutrition |
| --- | --- |
|  |  |
